# Peripheral Lymphadenopathy in Children: Cytomorphological Spectrum and Interesting Diagnoses

**DOI:** 10.5146/tjpath.2021.01537

**Published:** 2021-09-15

**Authors:** Arti Khatri, Nidhi Mahajan, Sonali Malik, Kanika Rastogi, Parveen Kumar, Diganta Saikia

**Affiliations:** Department of Pathology, Chacha Nehru Bal Chikitsalaya, Delhi, India; Medical Student, Gajra Raja Medical College, Gwalior, India; Department of Pediatric Surgery, Chacha Nehru Bal Chikitsalaya, Delhi, India; Department of Pediatric Medicine, Chacha Nehru Bal Chikitsalaya, Delhi, India

**Keywords:** Lymphadenopathy, Cytology, Child, Lymphomas, Mycobacterium

## Abstract

***Objective:*** Peripheral lymphadenopathy is a common complaint in the pediatric outpatient department. Fine needle aspiration cytology is the first investigation of choice with a high sensitivity for diagnosis but cytology may be challenging in some cases. The study was planned to study the cytomorphological spectrum and discuss a few interesting cases.

***Material and Method:*** 1890 paediatric subjects’ up to 12 years of age with significant peripheral lymph node enlargement and an adequate cytology specimen were included in the study. Inadequate aspirates were excluded.

***Results:*** The majority of children presented within 4-8 years of age with a male to female ratio of 1.7:1. The anterior cervical group was most commonly affected, followed by the posterior cervical, axillary and inguinal. Reactive lymphadenitis constituted the majority of the diagnoses, followed by Tuberculosis, acute suppurative, BCG-induced lymphadenitis, Kimura disease, Rosai-Dorfmann disease and Kikuchi-Fujimoto disease. Lymphomas and metastatic malignancies were less common, and mainly consisted of Hodgkin lymphoma, non-Hodgkin lymphoma, anaplastic large cell lymphoma, and Langerhans cell histiocytosis. Cytomorphological features of a few challenging and interesting cases have been discussed.

***Conclusion:*** Non neoplastic causes of lymphadenopathy predominate in the pediatric age group. A definitive diagnosis rests upon a complete clinical, radiological, microbiological, and cyto-histopathological correlation with the use of ancillary techniques wherever necessary.

## INTRODUCTION

Peripheral lymphadenopathy is a common clinical presentation in children. The etiology varies from benign to malignant; however, the commonest cause of lymphadenopathy in this age group is reactive (1). Sometimes systemic diseases or malignancies present with lymphadenopathies and therefore a better understanding of the differential diagnosis is important in guiding the clinician for timely evaluation and management. Developing countries like India more commonly have infectious causes of lymph node enlargement in comparison to developed nations where a neoplastic etiology is more common (2). The diagnosis predominantly rests upon fine needle aspiration cytology (FNAC) as it is cost-effective, minimally invasive, and easier to perform in smaller lesions. Biopsy, being an invasive procedure, is neither feasible nor advisable in all cases, thus making FNAC a cornerstone for near definitive diagnosis and further management in the pediatric population with this background, a retrospective study was conducted at a tertiary care pediatric hospital of North India to study the cytomorphological spectrum of peripheral lymphadenopathy and the underlying etiology while highlighting some interesting cases.

## MATERIAL and METHODS

This was a retrospective study conducted at Chacha Nehru Bal Chikitsalaya, a pediatric hospital in North India, for four years from January 2016 to January 2020. A total of 1890 cases were included in the study. All children less than 12 years of age with peripheral lymphadenopathy (cervical, axillary, supraclavicular, and inguinal) of significant size (more than 0.5 cm) were included in the study. Aspirates with non-diagnostic material were excluded. Informed consent was obtained for FNAC from either the parent or guardian as all subjects were less than 12 years of age. Approval from the Ethics Committee was waived off in view of the retrospective nature of the study.

A detailed history was taken including the duration and course of illness, and the associated clinical symptoms like fever, cough, weight loss, loss of appetite, history of respiratory tract infection, ear discharge, flaky itchy scalp, lice infestation, previous personal or family history of tuberculosis, local skin infections, immunization, and antibiotic therapy. The lymph nodes were examined for site, size, number, consistency, tenderness, mobility, and surface skin. FNAC was performed by cytopathologists using 22-, 23- or 24-Gauge needles. The aspirate was smeared on glass slides, air-dried for the Giemsa stain, and fixed in 95% ethanol for immunocytochemistry (ICC) in relevant cases. Cell blocks were prepared in cases suspicious for a neoplastic etiology. Special stains like the Ziehl-Neelsen stain, PAS, and mucicarmine were performed on smears and cell blocks whenever needed. Excision and histopathological confirmation were done in inconclusive cases or those with a strong suspicion of malignancy.

## RESULTS

A total of 1980 FNACs were performed for peripheral lymphadenopathy at the Department of Pathology. Of these, 90 (4.5%) were found inadequate. Histopathological examination was performed in 78 (4.1%) patients for a confirmatory diagnosis.

The patient age ranged between 0-12 years and was subdivided into three groups as 0-4 years, 4-8 years, and 8-12 years. 42% of the aspirates were done between 4-8 years of age; the youngest child was one month old whilst the oldest was 11 years 9 months. The study showed a slight male preponderance with a male:female ratio of 1.7:1. Tuberculous lymphadenitis showed a female preponderance (39.67%). Lymph nodes ranged in size from 0.5 to 5 cms. Cervical region involvement was most common, followed by axillary (Table 1). Generalized lymphadenopathy was seen in 2% of the cases.

**Table 1:** Characteristics of this study

**Total Number of Lymph Node FNAC**	2070
**No. of FNAC with adequate aspirate material**	1890
**Age Group Range (Mean age)**	1 month to 12 years (7 years)
**Male: Female ratio**	1.7 :1
**Sites of FNAC-**	
** Anterior Cervical**	948 (50.15%)
** Posterior Cervical**	453 (23.96%)
** Preauricular**	78 (4.12%)
** Submandibular**	108 (5.71%)
** Supraclavicular**	133 (7.03%)
** Axillary**	113 (5.97%)
** Inguinal**	57 (3.01%)
**Size of Lymph Nodes**	
** Smallest**	0.5 cm
** Largest**	5 cm
** Majority ranged between**	1-2 cm
**Total number of cases with histopathology available**	78
**Sensitivity**	89.47%
**Positive Predictive Value**	97.14%
**Localised Lymphadenopathy**	98%
**Generalised Lymphadenopathy**	2%

Benign pathology (98.2%) outnumbered malignant involvement in children (1.8%). Amidst the former category, reactive lymphadenitis was the commonest cause (64%) followed by granulomatous (20.8%, Tuberculosis {11%}), acute suppurative (12.8%), BCG adenitis (3.2%), Kimura disease, Kikuchi-Fujimoto disease, and Rosai Dorfmann disease (0.2% each). The malignant lesions were composed of Hodgkin lymphoma (48%), non-Hodgkin lymphoma (21%), leukemic infiltrate (9%), metastasis, anaplastic large cell lymphoma, and Langerhans cell histiocytosis (0.6% each). Six cases of tuberculous lymphadenitis were missed on cytology due to sampling errors, and their diagnosis was established on histopathology.

Histopathological correlation was available in only 4.1% of the cases as the majority were reactive, which does not warranty an excision. These were mainly cases which were either suspicious for malignancy on cytology or those which persisted despite antibiotic therapy. A 100% correlation of malignant lesions was found on histopathology except for one false positive case.

During the thorough workup of cases, we came across some challenging cases, which proved to be an enriching learning experience and are discussed below:

### Case 1

A three-year-old boy presented with bilateral cervical lymphadenopathy. FNAC from the node showed features of Non-Hodgkin’s Lymphoma, which on ICC turned out to be positive for T-cell markers (CD 3, CD 5). After 20 days, a review was requested, considering the lymph nodes had disappeared, but the reviewed diagnosis remained the same. After one month, the patient turned up again with lymphadenopathy for which excision was done. Histopathology showed T-cell lymphoma. Detailed history revealed that the patient received a course of oral steroids outside, following which the lymph nodes had disappeared.

### Case 2

**Figure 1:**
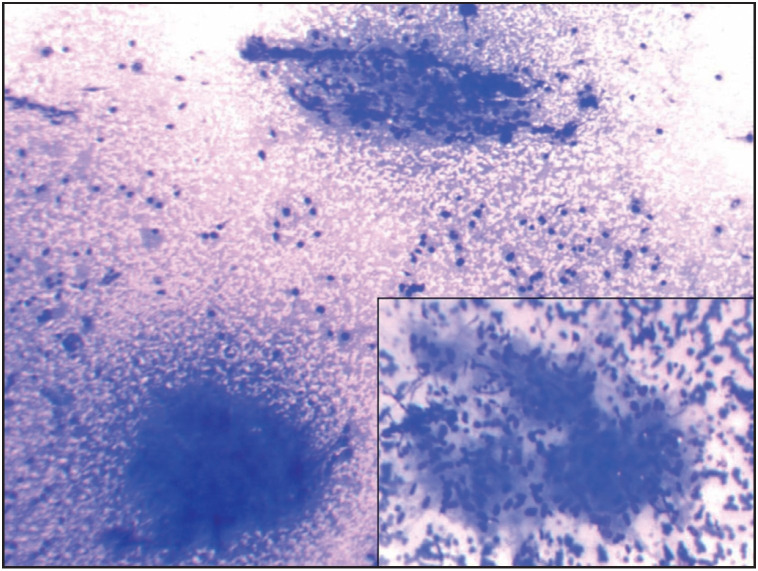
Streptococcus pyogenes induced lymphadenopathy, characterized by elongated cells, neutrophils and necrotic background (Giemsa; x100). Inset shows high power view of the epithelioid like cells (Giemsa; x400).

A two-year-old boy presented with posterior cervical lymphadenopathy for three weeks. Cytology showed presence of a reactive lymphoid population with focal areas of necrosis and clusters of elongated cells, which did not qualify as epithelioid cells (Figure 1). Microbiological examination of the aspirate was positive for Streptococcus pyogenes. Following this, the literature was reviewed and revealed that lymphadenopathy induced by Streptococcus pyogenes showed a similar morphology. His lymphadenopathy resolved post antibiotic therapy.

### Case 3

**Figure 2:**
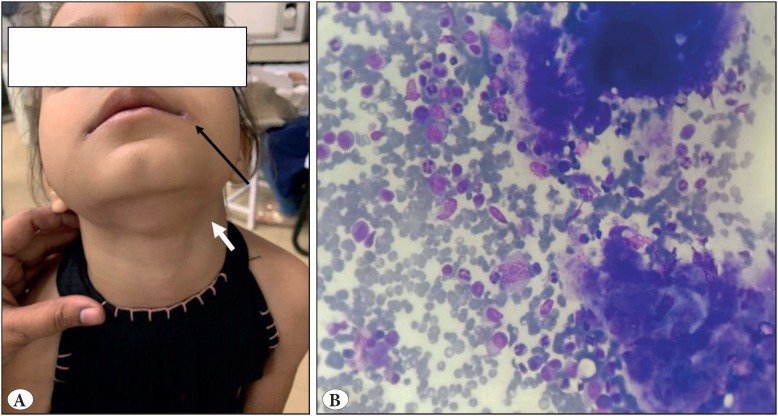
**A)** Lesion at angle of mouth (black long arrow) and left cervical lymphadenopathy (white short arrow). **B)** Smears show polymorphous population of reactive lymphoid cells, plasma cells, neutrophils, macrophages and foci of necrosis (Giemsa; x400).

A four-year-old girl presented with left-sided cervical lymphadenopathy and a lesion on the angle of the mouth (Figure 2), clinically suspicious of Kawasaki’s disease. Cytology smears showed polymorphous reactive lymphoid cells, plasma cells, neutrophils, and macrophages, along with few mitotic figures and focal areas of necrosis (Figure 2). These features were consistent with Kawasaki-associated lymphadenopathy.

### Case 4

**Figure 3:**
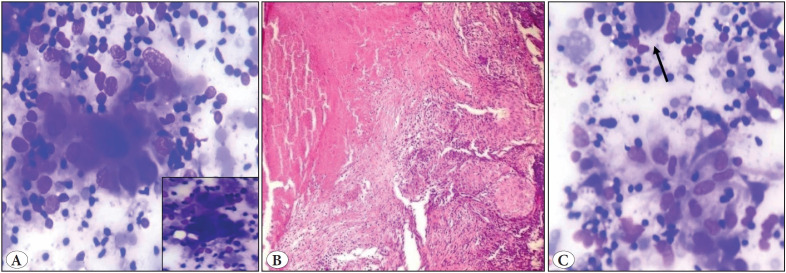
**A)** Cytology smears showing bizarre looking hyperchromatic cells (Giemsa; x100). Inset: showing atypical cell. **B)** Excision specimen showing caseous necrosis and epithelioid cell granulomas (H&E; x100). **C)** Review of smear showed rare ill formed epithelioid cell granulomas along with scattered atypical looking histiocytes (arrow) (Giemsa; x100).

A two-year-old girl presented with an enlarged left supraclavicular lymph node and significant weight loss. FNAC showed the presence of singly scattered and tiny clusters of atypical cells, which were large with high N: C ratio, round to eccentric nucleus, and prominent nucleoli (Figure 3). Metastasis was suspected, and radiological workup advised to look for the primary. However, the excised lymph node showed features of granulomatous lymphadenitis (Figure 3). Cytology slides were then reviewed and showed activated histiocytes with occasional tiny clusters of epithelioid cells (Figure 3). This finding emphasized the fact that activated histiocytes can sometimes mimic atypical cells.

### Case 5

**Figure 4:**
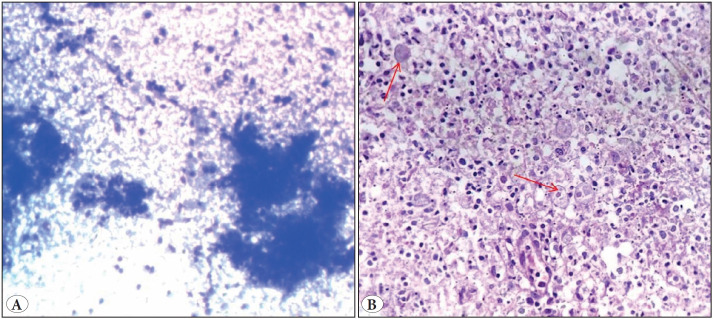
**A)** Cytology smears show diffuse necrosis (Giemsa; x100). **B)** Lymph node specimen showed amoebic trophozoites amidst background liquefactive necrosis (arrow) (H&E; x100).

A ten-year-old boy presented with right inguinal lymphadenopathy. FNAC showed diffuse necrosis (Figure 4). Microbiological studies were advised for ruling out tuberculosis, which turned negative. However, the patient’s condition worsened due to frequent abdominal pain, and excision of the inguinal node was advised, which showed extensive liquefactive necrosis with the presence of numerous amoebic trophozoites (Figure 4).

### Case 6

**Figure 5:**
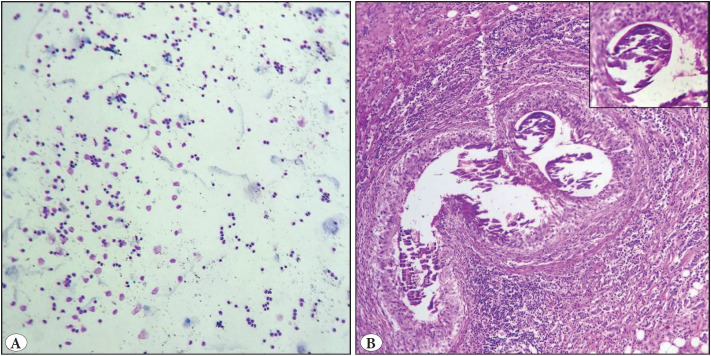
**A)** Somewhat fluid aspirate with mature lymphocytes (Giemsa; x400). **B)** Lymph node showing adult filarial worm within sinuses, inset- high power view of the worm (H&E; x100).

A 10-year-old boy presented with right-sided inguinal swelling. FNAC yielded somewhat fluid aspirate, which showed mature lymphocytes in a somewhat fluid and necrotic background (Figure 5). An impression of lymphangioma versus liquefactive necrosis was made. Ultrasound was performed, and showed a hypoechoic cystic lesion. The lymph node was excised and showed an adult filarial worm within the sinuses (Figure 5).

**Case 7:** A six-month male was brought with fever and a swelling on the left hand for five days. History revealed frequent episodes of fever, cough, and diarrhea. On examination, he was febrile with tachypnea, tachycardia, and hepatosplenomegaly. X-ray of the hand showed a lytic lesion in the 1st and 2nd metacarpals. FNA was done from the hand swelling with a clinical suspicion of Langerhans cell histiocytosis, congenital syphilis, and hematological malignancy. Smears showed diffuse sheets of histiocytes with negative ghost images (rod-shaped unstained structures) intracellularly and extracellularly in an inflammatory background. These images stained positive with ZN stain. On re-examination, there was an active BCG scar and left axillary lymphadenopathy. FNAC from both the left hand and the axillary swellings showed similar cytological features. Similar bacilli were seen in skin biopsy and bone marrow aspiration (BMA). The serum immunoglobulin profile done at this time revealed low IgM. AFB Culture was positive and Genotype MTBDR plus confirmed these bacilli as *Mycobacterium bovis*. Though antituberculous therapy (ATT) was started, the patient succumbed to death. This case has already been published and may be referred to for images (3).

## DISCUSSION

Lymphadenopathy is a common clinical manifestation in the pediatric age group. It may be a part of normal age-related physiology or in response to any local or generalized infection in the body, autoimmune disorders, or malignancy. Most children in the first decade of life experience primary or secondary lymphadenopathy at one point in time. Localized or generalized lymphadenopathy is the result of antigenic stimulation by pathogens or tumor cells. FNAC plays an important role in diagnosing lesions and masses at different sites as it is an easy, safe, rapid, and cost-effective tool. Generalized lymphadenopathy is defined as the enlargement of two or more groups of non-continuous groups of lymph nodes. It is worrisome as it results from of systemic illnesses like infections (viral, bacterial, fungal and protozoan), malignancies, autoimmune diseases, drug reactions, histiocytic disorders, disseminated neoplastic diseases, and storage disorders. In contrast, localized lymphadenopathy is usually the result of infections in the lymph node draining areas. The spectrum of lymphadenopathy is also variable amidst regions and even countries (4). Acute upper respiratory tract infections, cutaneous infections, and Tuberculosis are common causes of lymphadenopathy in developing countries like India and Africa, whereas autoimmune disorders and neoplasms are usually the cause of peripheral lymphadenopathies in developed countries where the rate of infections is relatively low (1). There is scant literature to support this evidence, especially from developed nations.

The sample inadequacy rate is higher in children than adults due to uncooperative, apprehensive behaviour and small-sized swellings. The unsatisfactory rate in the present study was 4.5% compared to other studies where the rate of unsatisfactory smears was 4.6% to 15% and was mainly due to smaller sized swellings (5,6). 98.2% of the cases were benign in etiology, and this data is similar to the studies by various authors (5,7,8). Amidst the benign causes, nonspecific reactive lymphadenitis was the most common (65.14%) followed by granulomatous (21.17%), which is also consistent with other studies (5,8,9). However, the percentage of reactive cases is higher in the present study when compared to studies done by Janagam and Atla (47.5%) but that can be explained by the wider age range and smaller sample size (10).

The anterior cervical group of lymph nodes were most commonly affected as any infection in the surrounding area like the oral cavity, ears, nose, and paranasal sinuses drain into these nodes. However, supraclavicular lymph nodes of any size are significant as they are almost always associated with an underlying pathology like metastasis or granulomatous disorders.

**Figure 6:**
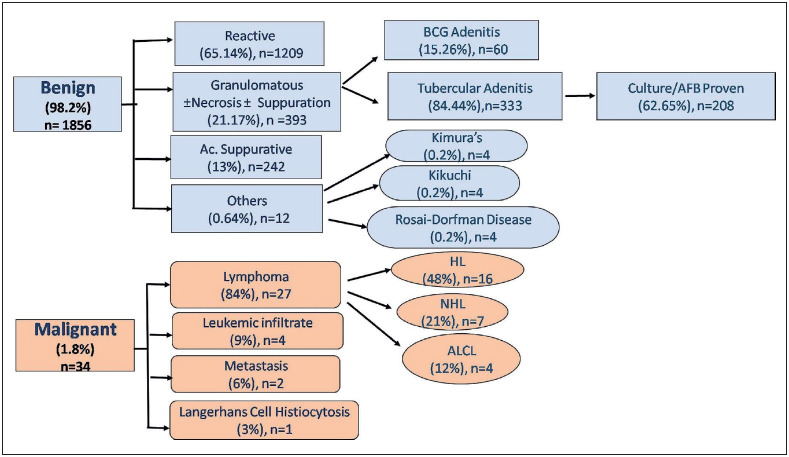
Schematic diagram showing the spectrum of paediatric lymphadenopathy in this study.

**Figure 7:**
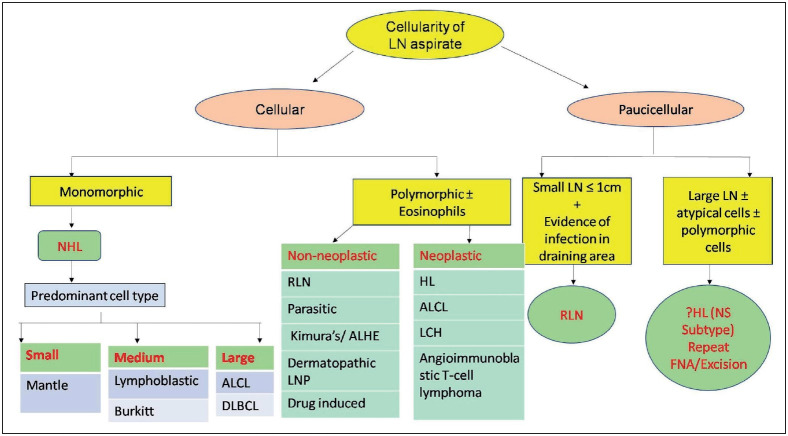
Systematic approach to diagnosis of enlarged lymph nodes. **LN:** Lymph node, **NHL:** Non Hodgkin lymphoma, A**LCL: A**naplastic large cell lyphoma, **DLBCL:** Diffuse large B cell lymphoma, **RLN:** Reactive lymph node, **LNP:** Lymphadenopathy, **HL:** Hodgkin lymphoma, **NS:** Nodular sclerosing, **LCH:** Langerhans cell histiocytosis

The cytological results in benign and malignant lesions sometimes show significant overlapping features, making a definitive diagnosis on cytology alone difficult. Thus, a systematic approach becomes handy in reaching a correct diagnosis and prevents the child from undergoing invasive procedures like biopsy (Figure 6 Figure 7).

Ultrasound-guided FNAC should be preferred over blind FNAC in deep seated and large sized nodes as the most suspicious part of the node can be sampled for an accurate diagnosis. Whenever there is doubt of metastatic deposits based on physical examination and imaging techniques, guided FNAC should be performed (11).

Any significant lymphadenopathy that does not subside, persists, or increases in size and is more than two weeks in duration requires thorough investigations7. A detailed clinical history about steroid administration is also imperative, considering the transient resolution of nodes post steroid therapy, especially in lymphoma cases. Transformed histiocytes and epithelioid cells in making sometimes show a high N:C ratio and can masquerade neoplastic cells on cytology. In infants, left sided axillary and lower cervical lymphadenopathy warrants careful examination of the BCG vaccination site.

In conclusion, reactive lymphadenitis is the commonest cause for lymph node enlargement in children. Features warranting active workup include a size more than two cms, hard consistency, matted nature, generalized or supraclavicular region involvement, and being nonresponsive to therapy for more than two weeks. Diagnosis of any form of peripheral lymphadenopathy in children requires a thorough clinical, radiological, microbiological, and cyto-histopathological correlation with the use of ancillary techniques whenever necessary.

## Conflict of INTEREST

The authors declare no conflict of interest.

## FUNDING

None
